# Ixodid tick infestation among camels (*Camelus dromedarius*) in Ethiopia: a systematic review and meta-analysis

**DOI:** 10.1186/s12917-026-05637-y

**Published:** 2026-06-17

**Authors:** Indiris Abdu, Anchiye Getachew, Abayeneh Girma

**Affiliations:** Department of Biology, College of Natural and Computational Sciences, Mekdela Amba University, Gimba, Ethiopia

**Keywords:** Ectoparasite, Tick infestation, Ixodid tick, Camel, Ethiopia

## Abstract

**Introduction:**

Ticks are major ectoparasites that significantly impact camels in Ethiopia. The local climate facilitates widespread infestation, particularly among camels, which are vital resources in East Africa. This study investigated pooled infestations of ixodid ticks in Ethiopian camels.

**Methods:**

In accordance with the PRISMA (Preferred Reporting Items for Systematic reviews and Meta-Analyses)-2020 guidelines, a comprehensive search was performed across multiple electronic databases, including Scopus, ScienceDirect, African Journal Online, PubMed, Web of Science and the Wiley Online Library. The Joanna Briggs Institute (JBI) checklist for prevalence data was used to assess the quality of included studies. Statistical examination was executed via Stata v.14. To calculate the overall infestation rate, a random-effects model was applied and established a 95% confidence interval for the data. To assess the consistency of the data, heterogeneity was measured using the I^2^ statistic, with further exploration conducted via Galbraith plots and subgroup and sensitivity analyses. To evaluate the presence of potential publication bias, the study utilized visual inspections of funnel plots complemented by the statistical rigor of Egger’s test. Ultimately, 11 cross-sectional studies met the inclusion criteria, totaling 4,895 animals (camels) across six regions of Ethiopia.

**Results:**

The meta-analysis in this review revealed an overall pooled infestation rate of ixodid tick infestations of 85.68% (95% CI: 78.29–93.06). Regional analysis revealed that the greatest percentage of infestations was in Tigray (96.6%), Dire Dawa (94.0%) and Afar (90.2%), whereas the lowest percentage of infestations was recorded in the Amhara region (55.2%). The taxonomic identification revealed the presence of three tick genera, specifically *Rhipicephalus (Boophilus)*, *Amblyomma*, and *Hyalomma*. *Rhipicephalus (Boophilus)* was the most dominant genus, with an infestation rate of 56.44%, whereas *Amblyomma* was the least common (14.65%).

**Conclusion:**

The current study revealed that infestations decreased from 90.18% from 2013 to 2019 to 82.03% from 2020 to 2025. In Ethiopia, ixodid ticks represent a major category of ectoparasites that persistently compromise the health and economic productivity of camel populations. Tick control actions must target the most common genera and the most affected geographic regions. Future research should shift toward investigating breed-specific susceptibility and the development of sustainable, integrated control strategies.

## Introduction

Camels possess a specialized suite of biological and physiological adaptations that enable them to thrive within the world’s most demanding arid and semiarid environments [1], feed on limited vegetation and tolerate water shortages. The arid lowlands of the Horn of Africa and its neighbors—specifically Somalia, Sudan, Ethiopia, Kenya, and Djibouti— are known for camel rearing [2]. Camels contribute approximately 6.3% of the total livestock production in eastern Africa. Camels are reared mainly in the eastern arid lowlands of Ethiopia in regions such as Tigray, Afar, Somali, Borena, and Dire Dawa. In Ethiopia, camels are primarily managed within pastoral and agro-pastoral frameworks, where they serve as a critical pillar of food security by providing a dependable supply of milk and meat, as well as essential transport services [3].

Despite Ethiopia’s major camel husbandry nations, the sector is significantly hindered by several constraints—most notably ectoparasite infestations and infectious diseases—which are further exacerbated by a lack of comprehensive veterinary care. Both endoparasites and ectoparasites, specifically ticks and mites, exert significant pressure on animal health, resulting in diminished productivity and overall physiological performance [3, 4]. Ticks are obligatory ectoparasites and hematophagous arthropods that infect many animal species [5]. The taxonomic classification of ticks identifies two primary families: Ixodidae, commonly known as hard ticks, and Argasidae, or soft ticks. The Ixodidae family is the largest and most common tick that predominantly infests camels [6]. For example, with over 20 distinct species of Ixodid ticks documented as infesting camels [7]. The genus *Hyalomma* is a common tick group that infests camels; specifically, *Hyalomma dromedarii* is the main and major camel-specific species worldwide [8].

Ticks serve as critical biological vectors for a variety of zoonotic diseases, which constitute a primary driver of global livestock morbidity and mortality, significantly undermining national economies and causing extensive losses within the sector [9]. Tick infestation results in a wide range of problems, including skin swelling and small wounds from bites, irritation, tick-borne disease concerns, blood loss, mild to severe anemia, loss of appetite, weight loss, increased death and vulnerability to other infectious diseases, and decreased productivity. Certain tick species secrete potent neurotoxins during feeding that directly impair the nervous system and muscular coordination, often resulting in progressive paralysis and, if left untreated, animal mortality [10].

A comprehensive understanding of the prevalence and epidemiological characteristics of tick-borne diseases—particularly the transmission dynamics of the vectors—is essential for the development of strong prevention and control frameworks [11]. In Ethiopia, various scattered and independent research has been performed on the infestation rate with associated determinants for ticks in different livestock, such as cattle, camels, and small ruminants. Furthermore, two systematic reviews and meta-analyses reported the combined infestation rates of hard ticks in cattle [12] and small ruminants [13]. To date, no comprehensive systematic review or meta-analysis has been conducted to quantify the aggregate prevalence of tick infestations in camels within Ethiopia, representing a significant gap in the current veterinary literature. Consequently, this review was designed to synthesize and evaluate the pooled infestation of ixodid tick among dromedary camels (*Camelus dromedarius*) across Ethiopia.

## Methodology

This systematic review and meta-analysis was conducted in strict adherence to the PRISMA (Preferred Reporting Items for Systematic Reviews and Meta-Analyses) statement to ensure methodological transparency and rigor [14].

### Search strategy

The main databases used to search for all published articles for this study were the Wiley Online Library, PubMed, Web of Science, African Journals Online, ScienceDirect and Scopus, as well as sources such as Google Scholar. All studies published from 2013–2025 were included. To identify relevant research articles, the following keywords and phrases were utilized, either independently or in conjunction with Boolean operators (“OR” and “AND”): “infestation rate”, “Epidemiology”, “Tick burden”, “Camel,” “Tick”, “Hard tick”, “Ixodid tick,” “tick genera,” “tick infestation,” “*Rhipicephalus* (*Boophilus*),” “*Amblyomma*,” “*Hyalomma*”, “Ethiopia”, “Amhara”, “Oromia ”, “Somali”, “Afar, “Dire Dawa”, “Tigray ”, “Benishangul-Gumuz”, “Southern nation, nationalities and peoples region (SNNPR)”, and “Harari and Gambella”. The comprehensive literature search was conducted over a three-month period, spanning from August to October 2025.

### Inclusion and exclusion criteria

To ensure the selection of high-quality and pertinent data, a rigorous set of inclusion and exclusion criteria was established to filter the search results.

Inclusion criteria

- ✓ Original articles with observational study designs reported in Ethiopia among camels
- ✓ Articles that reported number of examined camels, number of infested camels, or infestation rate
- ✓ Articles published between 2013 and 2025, with full texts available in English
- ✓ Studies that assessed ixodid ticks and identified or grouped them at the genus level

Exclusion criteria

- Studies reporting only the knowledge, attitudes, and practices (KAPs) of veterinarians or camel owners regarding ixodid ticks (without original tick infestation or identification data)
- Studies conducted on domestic animals other than camels
- Studies focusing on soft ticks (Argasidae)
- Studies published before 2013
- Nonoriginal studies

### Data extraction

A standardized data extraction form was developed in Microsoft Excel 2021. Relevant data were extracted from each eligible full-text article and entered into the data extraction form following the PRISMA-2020 guidelines. The data extracted incorporated the following: first researcher’s name, year of study, study region and design, sampling method, number of examined camels, number of infested camels, infestation rate, total collected ticks, and each tick genus collected. All three authors (InA, AnG, and AbG) reviewed the collected articles, applying the agreed-upon inclusion and exclusion criteria. When two authors did not agree, a third author stepped in. All differences were then resolved through discussion and consensus.

### Quality assessment tool

The methodological rigor of the selected research was evaluated using the Joanna Briggs Institute (JBI) Critical Appraisal Checklist specifically designed for studies reporting prevalence data [15]. The criteria enable a systematic evaluation of key questions, such as sample size, the sampling frame and method, study setting, participant recruitment, response rate, measurement of condition (tick identification), and appropriate statistical analysis.

Each of the nine checklist items was recorded as “yes” (1), “no/ unclear " (0),“, yielding a total quality value ranging from 0–9. Articles were incorporated in the present review if they achieved a minimum quality score of five or higher, indicating that more than half of the checklist criteria were met. This standardized stratification facilitated a transparent evaluation of the risk of bias across all included studies, ensuring the integrity of the meta-analytical results.

The literature selection and quality assessment were conducted independently by two reviewers (IA and AnG), with any discrepancies resolved through consensus-based discussion prior to final study inclusion.

### Data analysis

The primary response variable was tick infestation among camels, operationalized as the infestation rate calculated for each study as the number of camels infested with ixodid ticks divided by the total number of camels examined multiplied by 100. After data extraction into Microsoft Excel 2021, the data were transferred to Stata v. 14 for examination. Owing to anticipated heterogeneity among studies, a meta-analysis random-effects model was employed to synthesize the pooled infestation rate and its 95% confidence interval (CI). The chi-square contribution for each tick genus is calculated as the square of the difference between the observed and expected count divided by the expected count. For simplicity and computational convenience, meta-analyses commonly assume a normal distribution in random effects models. Accordingly, both the within-study sampling errors and the among study effects were assumed to be normally distributed. The inverse variance method was used to assign study weights, incorporating both within-study variance and among study variance (τ²), with the REML (restricted maximum likelihood) method used to estimate τ². Heterogeneity was evaluated via the Galbraith plot, Cochrane Q test, and the I^2^ statistic values range from 0% to 100% and interpreted 0%–25% (low), 25%–50% (moderate), 50%–75% (substantial), and 75%–100% (considerable heterogeneity) [16]. For the Galbraith plot, studies falling outside the 95% confidence intervals (± 2 on the standardized effect size scale) were considered contributors to statistical heterogeneity. Subgroup analyses on the basis of region, number of examined camels, publication year, and tick genera were conducted to investigate the sources of heterogeneity. Funnel plots and Egger’s regression test were used to assess publication bias, where a p value less than 0.05 shows evidence of statistically significant bias [17].

## Results

### Selection of studies

**Fig. 1:**
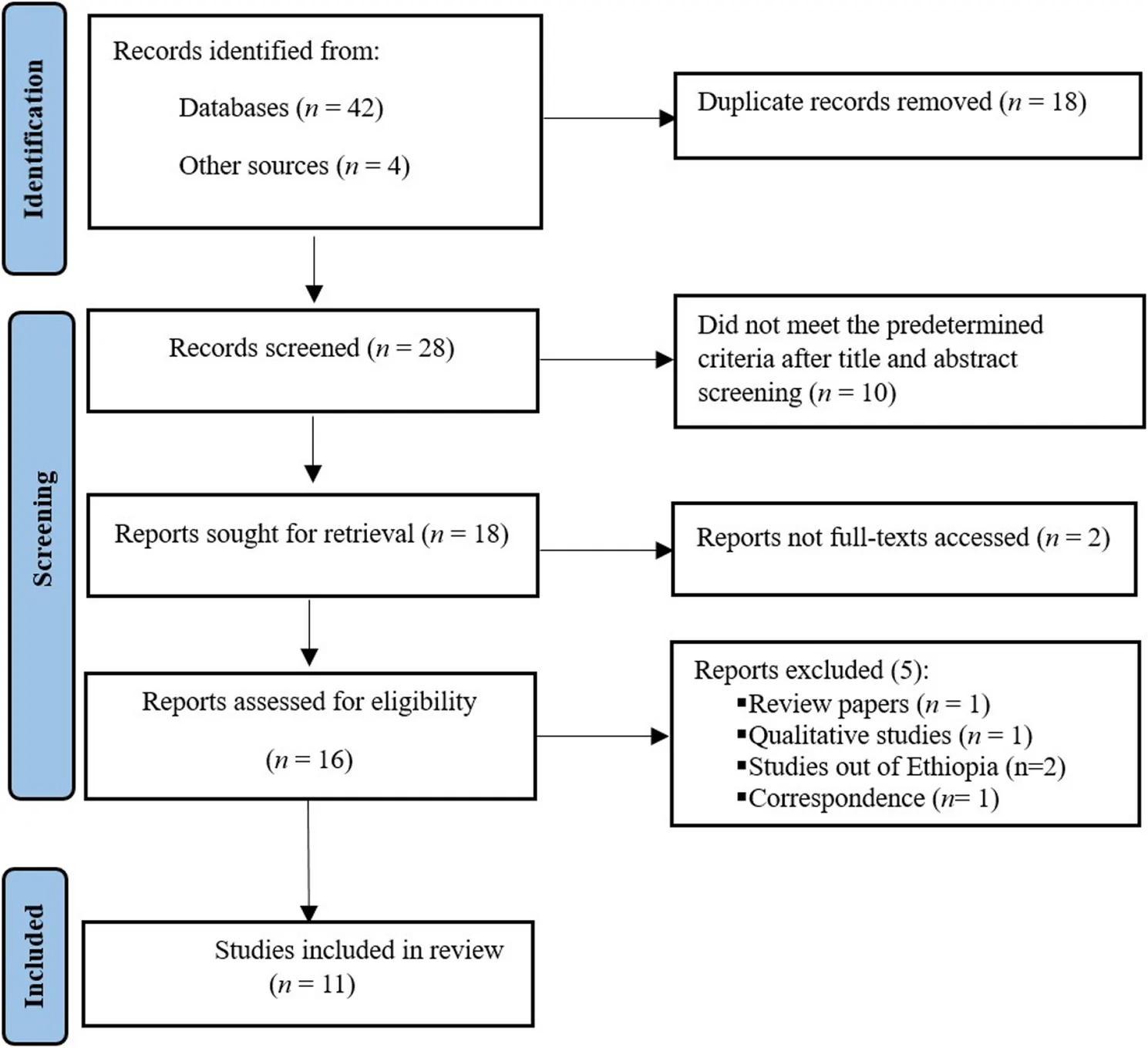
PRISMA flow diagram illustrating the systematic literature search and selection process

An initial total of 46 records were identified across electronic databases and subsequently exported to EndNote X9 (Clarivate Analytics) for systematic reference management and duplicate removal. To ensure the integrity of the dataset, duplicate records were identified and eliminated using a combination of automated software functions and rigorous manual cross-referencing, leading to the exclusion of 18 articles. The remaining titles and abstracts were subjected to an independent screening process conducted by the authors in accordance with the predefined eligibility criteria, resulting in the exclusion of an additional 10 studies. A rigorous full-text assessment was then performed on the remaining articles, where two were removed because they failed to meet specific eligibility requirements. Furthermore, five studies were excluded for the following reasons: they were review articles (*n* = 1), qualitative studies (*n* = 1), studies conducted outside of Ethiopia (*n* = 2), or correspondence studies (*n* = 1). Any discrepancies during the selection process were resolved through consensus-based discussion. Any remaining disagreements during the selection phase were reconciled through consensus-based discussion, ensuring a unified approach to study eligibility. Following this rigorous screening process, a final total of 11 studies met all predefined inclusion criteria and were incorporated into the systematic review and meta-analysis (Fig. 1).

### Characteristics of the included studies

A simple random sampling technique was employed in all eligible cross-sectional studies included in this review. These 11 articles originated from six different Ethiopian regions, with the highest concentration of research located in the Oromia region (*n* = 4) [18–21]. The Somali region [22, 23] and the Afar region [24, 25] follow each of which two studies are produced. Dire Dawa [26], Tigray [27] and Amhara [28] contributed to only one study each. With respect to the temporal distribution of the literature, six of the included studies were published after 2020, whereas five were published before 2020. The studies were also categorized by cohort size: three used a sample size exceeding 384, whereas eight used a sample size of exactly 384. Analysis of the identified literature revealed that the cumulative infestation rate of Ixodid ticks in camels exhibited substantial variability, spanning from 55.21% to 97.7%. Within these individual surveillance efforts, the number of examinations ranged from 384 to 813 camels per study, as detailed in Table 1.

**Table 1:** General characteristics of the studies included in the systematic review and meta-analysis of ixodid tick infestations among camels in Ethiopia

Author	Year	Region	Sample size	Number of animals infested	Infestation rate (%)	Total ticks collected	Quality
Yirsa et al.	2025 [28]	Amhara	384	212	55.21	1008	9
Desta	2025 [24]	Afar	384	349	89.1	1424	9
Ahmed et al.	2024 [18]	Oromia	384	295	76.8	3145	9
Ahmed et al.	2024 [19]	Oromia	384	314	81.7	4850	9
Feki et al.	2021 [25]	Afar	813	573	91.10	–	7
Elias et al.	2020 [20]	Oromia	384	374	97.4	4417	9
Megersa et al.	2019 [21]	Oromia	560	547	97.7	6436	7
Hussen & Agonafir	2018 [22]	Somali	384	318	82.8	5,090	9
Feyer et al.	2017 [23]	Somali	450	354	78.6	6793	7
Kiros et al.	2014 [27]	Tigray	384	371	96.6	15,723	8
Taddese & Mustefa	2013 [26]	Dire Dawa	384	361	94	11,774	9

Infestation rate (%) = (number of animals infested/ number of animals examined) × 100. A dash (–) indicates data not reported. The study years correspond to citation numbers in brackets

### Distribution of major ixodid tick genera collected from camels in Ethiopia

The aggregate entomological data reveals a substantial parasite burden across the reviewed literature, with a cumulative total of 60,660 Ixodid ticks collected and identified. The genus *Rhipicephalus (Boophilus)* was the most common (34,238 ticks; 56.44%), followed by *Hyalomma* (17,534 ticks; 28.91%) and *Amblyomma* (8,888 ticks; 14.65%). The results of the chi-square (χ²) goodness-of-fit test demonstrated that the distribution of ticks among the three genera was significantly different from an equal distribution (χ² = 16,425.9, df = 2, *p* < 0.001). The implementation of post hoc pairwise comparisons with a Bonferroni adjustment confirmed that *Rhipicephalus (Boophilus)* was significantly more abundant than both *Hyalomma* (*p* < 0.001) and *Amblyomma* (*p* < 0.001), and *Hyalomma* was markedly more abundant than *Amblyomma* (*p* < 0.001) (Table 2).

**Table 2:** The distribution of ixodid tick genera collected from camels in Ethiopia

Author	Year	Tick Genera Collected			
		Hyalomma	Amblyomma	Rhipicephalus (Boophilus)	Total collected Ticks
Yirsa et al.	2025 [28]	457	274	277	1008
Desta	2025 [24]	683	146	595	1424
Ahmed et al.	2024 [18]	1016	694	1435	3145
Ahmed et al.	2024 [19]	1710	1050	2090	4850
Elias et al.	2020 [20]	414	164	3839	4417
Megersa et al.	2019 [21]	695	993	4748	6436
Hussen & Agonafir	2018 [22]	1761	1146	2183	5090
Feyer et al.	2017 [23]	2342	1031	3420	6793
Kiros et al.	2014 [27]	3981	1747	9995	15,723
Taddese & Mustefa	2013 [26]	4475	1643	5656	11,774
Total		17,534	8888	34,238	60,660
Tick distribution (%)		28.91	14.65	56.44	100
95% CI		(27.55 − 29.27)	(13.37 − 15.93)	(55.05 − 57.83)	N/A
χ² test (df = 2)		356.8	6,350.8	9,718.3	χ² = 16,425.9

Tick distribution (%) for each genus = (number of ticks of that genus/total ticks collected across all studies) × 100. A total of 60,660 ticks were collected across all the studies. The study years correspond to citation numbers in brackets. *Rhipicephalus* (*Boophilus*) refers to ticks of the subgenus *Boophilus*. *χ²* = Σ [(observed frequency – expected frequency) ²/expected frequency]

CI= confidence interval. N/A = not applicable

### Meta-analysis

**Fig. 2:**
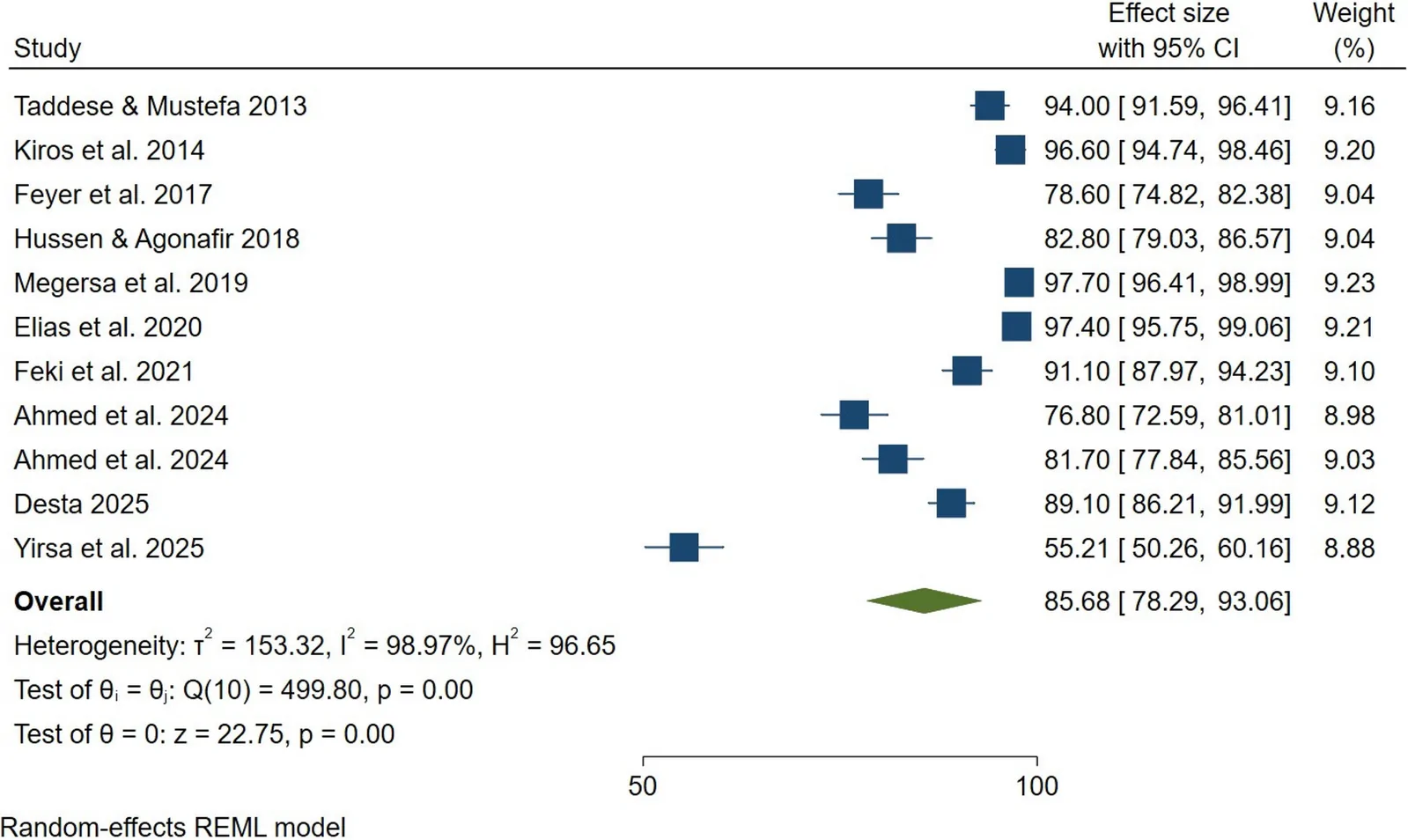
Forest plot of the pooled Ixodid tick infestations in Ethiopian camels. Individual study effect sizes are represented by squares; horizontal lines represent 95% CIs; and the diamond shows the pooled infestation (85.68%, 95% CI: 78.29–93.06) via a random-effects (REML) model. Heterogeneity: I² = 98.97%, Q = 499.80, *p* < 0.001

A random-effects meta-analysis model was employed to pool the infestation data, incorporating an intercept-only framework that assumes a normal distribution for study-specific random effects. The overall pooled infestation rate (response variable: tick infestations among camels) was 85.68% (95% CI: 78.29–93.06). The between-study variance was τ² = 153.32. The high observed heterogeneity (I² = 98.97%, Q = 499.80, df = 10, *p* < 0.001) confirmed the appropriateness of the random-effects model over a fixed alternative (Fig. 2).

### Subgroup analysis

The Tigray region had the greatest pooled infestation of ixodid ticks among the camels in the present review (96.60%; 95% CI: 94.74 − 98.46), followed by Dire Dawa (94.00%; 95% CI: 91.59 − 96.41) and the Afar region (90.20%; 95% CI: 87.90 − 92.14). However, the Amhara region had the lowest number of tick infestations among the camels (55.21%; 95% CI: 50.26 − 60.16). Comparative analysis categorized by number of camels examined indicated that studies utilizing more than 384 subjects reported a greater aggregate infestation (89.25%; 95% CI: 78.69–99.81) than studies with exactly 384 subjects (84.42%; 95% CI: 77.28–91.56). The pooled infestation rate of ixodid ticks in the years 2013–2019 (90.18%; 95% CI: 84.37–95.99) was greater than that in the years 2020–2025 (82.03%; 95% CI: 71.91–92.15) (Table 3).

**Table 3:** Subgroup analysis of ixodid tick infestation rates among camels in Ethiopia by region, sample size, and publication year

Variables	Characteristics	Included studies	Sample size	Infestation rate (95% CI)	I ^2^, *P*–value	*p* value (within subgroup)	*p* value (between subgroups)
Region	Dire Dawa	1	384	94.00 (91.59 − 96.41)	N/A	N/A	*p* < 0.001
	Tigray	1	384	96.60 (94.74 − 98.46)	N/A	N/A	
	Somali	2	834	80.70 (76.59 − 84.82)	58.0%, *p* = 0.123	*p* = 0.123	
	Oromia	4	1712	88.69 (81.13 − 96.25)	97.9%, *p* < 0.001	< 0.001	
	Afar	2	1197	90.02 (87.90 − 92.14)	0.0%, *p* = 0.358	*p* = 0.358	
	Amhara	1	384	55.21 (50.26 − 60.16)	N/A	N/A	
Sample size	384	8	3072	84.42 (77.28 − 91.56)	98.2%, *p* < 0.001	*p* < 0.001	*p* = 0.458
	> 384	3	1823	89.25 (78.69 − 99.81)	97.9%, *p* < 0.001	*p* < 0.001	
Publication year	2013–2019	5	2162	90.18 (84.37 − 95.99)	97.0%, *p* < 0.001	*p* < 0.001	*p* = 0.171
	2020–2025	6	2733	82.03 (71.91 − 92.15)	98.5%, *p* < 0.001	*p* < 0.001	
Total		11	4895	85.68 (78.29 − 93.06)	98.97%, *p* < 0.001	*p* < 0.001	N/A

I² denotes the percentage of heterogeneity

*CI* Confidence interval, *N/A* Not applicable

Moreover, formal subgroup difference testing (Q-between) revealed a statistically significant difference in pooled infestation across regions (*p* < 0.001), with the Amhara region showing markedly lower infestation (55.21%) than other regions (range: 78.60–97.70%). In contrast, no statistically significant differences were observed between studies with sample sizes of 384 versus > 384 (*p* = 0.458) or between studies published from 2013 to 2019 versus those published from 2020 to 2025 (*p* = 0.171), suggesting that neither sample size nor publication year significantly modified the pooled infestation estimate.

### Quality assessment results

The quality scores of the incorporated articles, which are based on JBI checklist (0–9 scale), are presented in Table 1. The scores ranged from 7 to 9, with a mean of 8.36 ± 0.92. Among the 11 included studies, 11 (100%) were categorized as high quality (7–9 points), indicating that more than half of the checklist criteria were met.

### Heterogeneity, publication bias, and sensitivity analysis

**Fig. 3:**
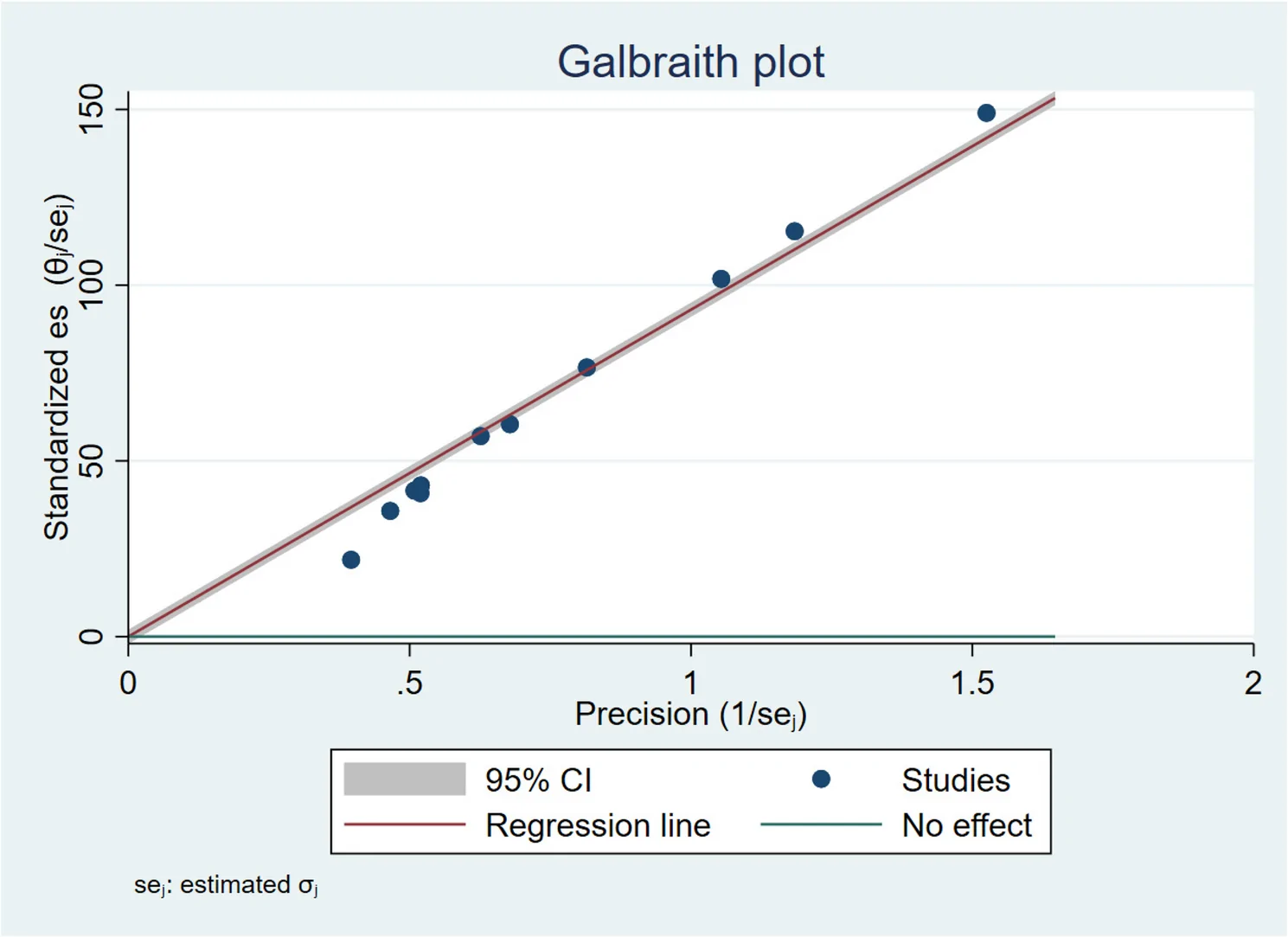
Galbraith plot evaluating heterogeneity in the study of ixodid tick infestations among camels in Ethiopia

The Galbraith plot visually assesses heterogeneity by plotting the standardized effect size (θ/σ) against precision (1/se). Under homogeneity, approximately 95% of articles are expected to be within the gray shaded region (approximately ± 2 standard deviations from the regression line). In Fig. 3, 9 of 11 studies fell outside these 95% confidence bands, indicating that the observed dispersion exceeds what would be expected by chance alone. This visual finding is consistent with the quantitative heterogeneity statistics (I² = 98.97%, Q = 499.80, *p* < 0.001), which exceed conventional thresholds for substantial heterogeneity (I² > 75%, Q test *p* < 0.10).

**Fig. 4:**
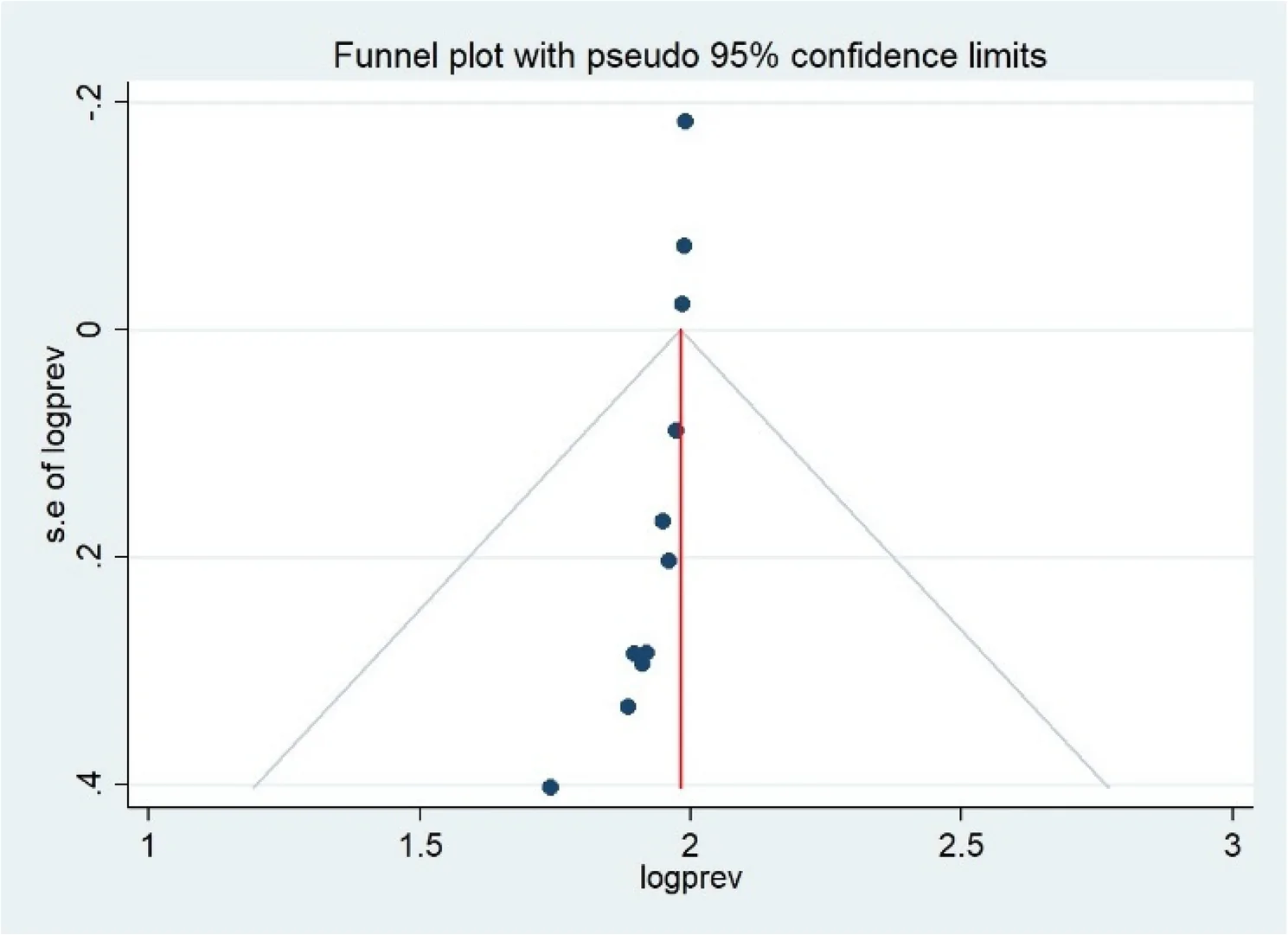
Funnel plot for the visual evaluation of potential publication bias

Publication bias of the funnel plot (Fig. 4) suggested asymmetry, with studies unevenly distributed around the pooled effect size (85.68%). Egger’s regression test of small-study effects (Table 4) formally confirmed the presence of statistically significant publication bias (bias coefficient = -15.07, 95% CI: -19.88 to -10.27, *p* = 0.00).

**Table 4:** Statistical assessment of small-study effects using Egger’s linear regression test for camel ixodid tick infestation data

Std_Eff	Coef.	Std. Err.	T	*P*>|t|	[95% Conf. Interval]
Slope	109.6331	2.567688	42.70	0.00	103.8246, 115.4416
Bias	-15.07221	2.1241	-7.10	0.00	-19.87726, -10.26717

*Coef* Coefficient, *Std. Err* Standard error, *T* t statistic test, *Conf. Interval* Confidence interval

**Fig. 5:**
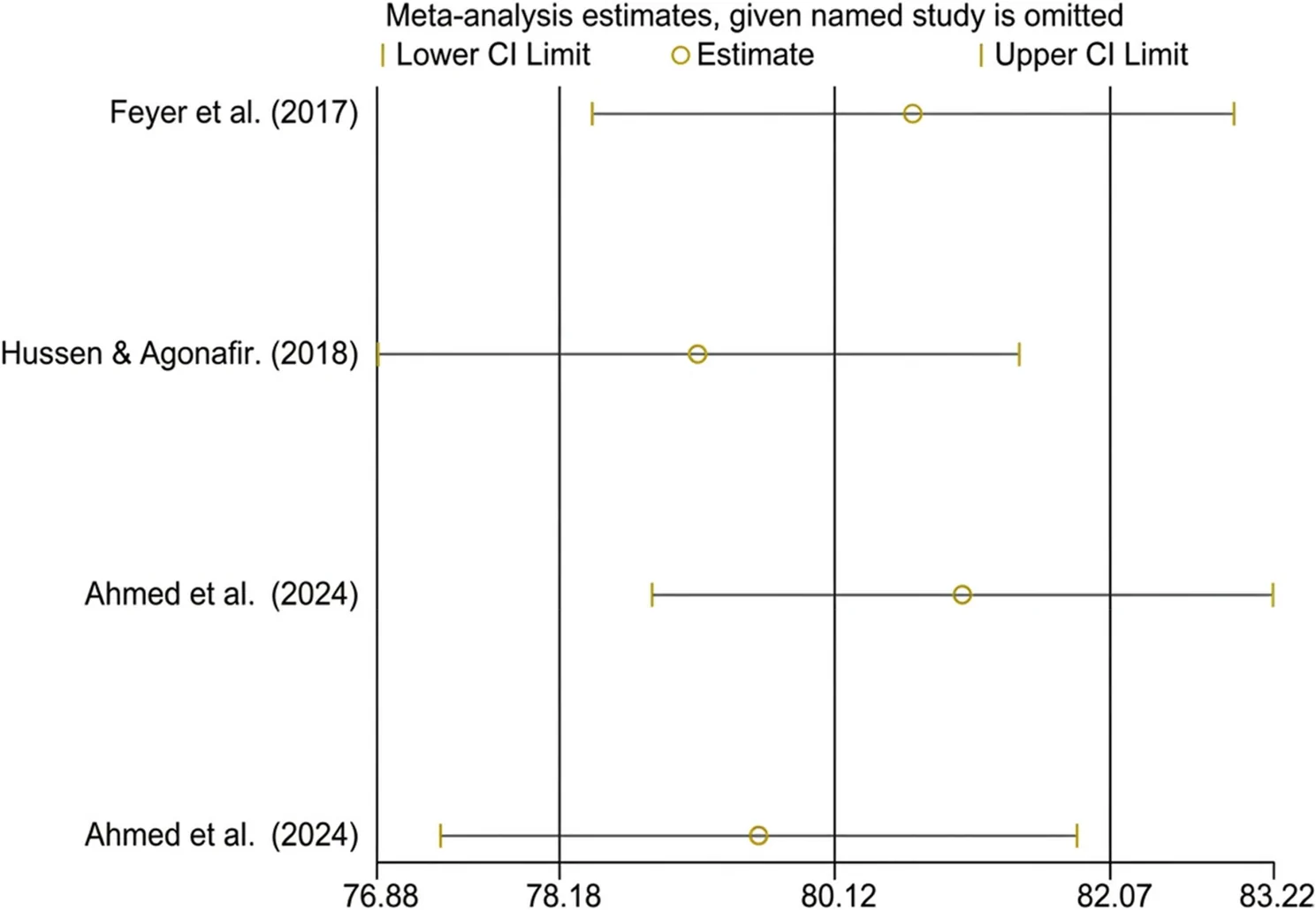
Sensitivity analysis of the pooled ixodid tick infestation rates among Ethiopian camels using the leave-one-out approach

The sensitivity (leave-one-out) analysis (Fig. 5) revealed that the overall estimate becomes similar at approximately 80% after each study was sequentially omitted, with values ranging from 78.18% to 83.22%. Notably, even the exclusion of the lowest (Yirsa et al., 2025: 55.21%) or the highest (Megersa et al., 2019: 97.70%; Elias et al., 2020: 97.40%) infestation rate studies did not shift the pooled estimate substantially outside the 78–83% range. This stability indicates that no study applied unnecessary influence and that the pooled estimate of approximately 80% is robust despite substantial heterogeneity across the included studies.

## Discussion

Ticks are the primary carriers of diseases that harm camels worldwide, posing a serious health concern for Ethiopian camels. This review was performed to estimate the pooled infestation of tick in Ethiopia. In this review, 4895 camels were examined, and 60,660 ticks were collected, yielding a pooled infestation rate of 85.68%.

The current findings were comparable to those of Mohsen et al. (85.5%) [29]. However, this figure was higher than those reported in Iran (53.9%) [30], Saudi Arabia (68.2%) [7] and Iraq (79.54%) [31]. However, the current result was less than those of reported from Hargessa, Somali, Africa (93.75%) [32] and Iraq (97.1%) [33]. The difference in findings might be because of differences in geography, husbandry practices, seasons, agroecosystems, animals’ healthcare practices and research designs, which may not be favorable to the survival and reproduction of camels [34].

Substantial statistical heterogeneity (I² = 98.97%, *p* < 0.001) was observed among the 11 eligible articles, due to variations in geography, season, sampling methods, and husbandry practices. The Galbraith plot confirmed dispersion beyond expectations of homogeneity, whereas the sensitivity analysis indicated that the overall estimate remained stable at approximately 80%, indicating a consistent central tendency despite considerable individual study variation. Egger’s regression test in the small-study effects was significant (*p* < 0.001), suggesting a potential publication bias. However, Egger’s test is influenced by between-study heterogeneity; given the high I² value, the significant result may partly reflect true differences in infestation rates rather than exclusively indicating publication bias. Therefore, while publication bias cannot be ruled out, funnel plot asymmetry should be interpreted cautiously, and the pooled estimate is best viewed as an average across diverse settings.

Among the different regions of Ethiopia, the highest percentage of infestations of ixodid ticks among camels was documented in Tigray (96.60%), followed by Dire Dawa (94.00%) and Afar (90.20%), whereas the lowest percentage of infestations was recorded in the Amhara region (55.21%). Regional variation may be related to agroecology, host density, and livestock management practices [35]. The highest burdens are found in arid and semiarid lowlands (Tigray, Dire Dawa, Afar), whereas a significantly lower infestation is recorded in more temperate or highland-adjacent areas (Amhara) [36]. The pooled infestation with a sample size exceeding 384 (89.25%) camels was greater compared to those utilizing a standard sample of examined camels 384 (84.42%). This could be because studies with larger sample sizes (more samples) often cover a wider geographical area and find a vast area of infestation that drives the infestation rate [37].

The systematic review identified and grouped three major tick genera—*Rhipicephalus (Boophilus)*, *Hyalomma*, and *Amblyomma*. Among the three identified tick genera, the most common was *Rhipicephalus* (*Boophilus*), at 56.44%, followed by *Hyalomma*, at 28.91%, whereas *Amblyomma* was the least common genus (14.65%; 95% CI 13.37 − 15.93). The tick genus *Rhipicephalus* (*Boophilus*), which is the most common among the camels in this study, contradicts studies from camel-rearing countries, where *Hyalomma* is often reported as the most common. The high infestation rate of *Rhipicephalus* is probably due to its opportunistic feeding nature and is common in the semiarid Rift Valley and the eastern lowlands of Ethiopia [38]. Additionally, *Boophilus* is now taxonomically classified as a subgenus within *Rhipicephalus*, previously treated as a separate genus, and is summarized as *Rhipicephalus* (*Boophilus*) in the current study. This may affect the current infestation rate. *Hyalomma* is the second most common genus and is the basis of camel parasitology [39]. The genus *Hyalomma* includes camel-specific hunters that actively move toward their hosts. In extremely arid desert conditions, *Hyalomma* is often the principal genus due to its superior ability to survive extreme dryness [40].

In the current review, the infestations of ixodid ticks among camels reduced from 90.18% from 2013 to 2019 to 82.03% from 2020 to 2025. This decreasing trend in pooled infestations could be because of improved breed variation, improved husbandry practices and availability of veterinary services [2].

Tick infestations and tick-borne illnesses may cause multifaceted financial losses, encompassing both immediate productivity deficits and secondary systemic complications. Direct losses are primarily attributed to disease-induced morbidity and mortality within the camel population [41]. Beyond the immediate threat of mortality, infested camels suffer from chronic conditions such as weight loss, hide degradation due to cutaneous lesions, and reduced draught capacity. Furthermore, long-term developmental delays, including impaired fertility and retarded maturation, pose significant challenges to the sustainability of pastoralist livestock systems [41–43]. Several approaches have been used in tick control, including cross-breeding, biological control methods, multifaceted tick control tactics, and the development of vaccines against tick antigens [44]. Compared with intensive systems (where livestock are kept in confined, controlled environments), environmental management, including the seasonal variation of infestations and prolonged rearing, increases the impact of infestations. Chemical spraying or dipping is the most traditional method of tick management [43].

### Limitations of the study

The potential limitations shall be considered during using the results of this review. Substantial heterogeneity (I² = 98.97%) was detected among the 11 incorporated articles, due to differences in geographical regions, seasonal factors, sampling methods, and camel management practices. Geographically, the studies were concentrated in only six Ethiopian regions, limiting their generalizability to the entire country. All included studies employed cross-sectional designs with simple random sampling, which are susceptible to uncontrolled confounding variables (e.g., age, sex, husbandry practices) that may influence reported infestation rates. The review restricted inclusion to studies published between 2013 and 2025 in English, potentially introducing temporal and language bias. Additionally, the significant Egger’s test (*p* < 0.001) suggests possible publication bias, although this finding may also reflect substantial between-study heterogeneity. Finally, this review focused exclusively on tick infestations and did not analyze specific risk factors such as: body condition score (BCS), age, sex, seasonality, herd size and management practice, representing an important area for future research.

## Conclusions

The present study revealed high rates of ixodid tick infestations among camels in Ethiopia (85.68%). The findings underscore the status of ticks as ubiquitous and economically vital ectoparasites affecting the Ethiopian dromedary population. In the current review, *Rhipicephalus* (*Boophilus*), with an infestation rate of 56.44%, and *Amblyomma*, with an infestation rate of 14.65%, were the most and least common ixodid tick genera recorded among camels in the country. Analysis of temporal trends revealed a moderate decline in infestation rates, decreasing from 90.18% from 2013 to 2019 to 82.03% from 2020 to 2025. Despite this decreasing trend, persistent high infestations continue to severely impact camel productivity and animal health in Ethiopia. Consequently, it is imperative to design and implement targeted tick control programs that reflect these current epidemiological findings. Moreover, longitudinal studies are immediately needed to elucidate specific predictors, the pathogenic role of various ixodid species, and the resulting economic implications for pastoralist livelihoods.

## Data Availability

The datasets used and/or analysed during the current study are available from the corresponding author on reasonable request.

## Abbreviations

CI: Confidence interval
Df: Degree of freedom
JBI: The Joanna Briggs Institute
KAP: Knowledge, attitudes, and practices
N/A: Not applicable
PRISMA: Preferred Reporting Items for Systematic reviews and Meta-Analyses
χ²: Chi-square test
